# Assessment of cardiovascular physiology using dobutamine stress cardiovascular magnetic resonance reveals impaired contractile reserve in patients with cirrhotic cardiomyopathy

**DOI:** 10.1186/s12968-015-0157-6

**Published:** 2015-07-18

**Authors:** Francisco Sampaio, Pablo Lamata, Nuno Bettencourt, Sophie Charlotte Alt, Nuno Ferreira, Johannes Tammo Kowallick, Joana Pimenta, Shelby Kutty, José Fraga, Michael Steinmetz, Paulo Bettencourt, Vasco Gama, Andreas Schuster

**Affiliations:** Cardiology Department, Centro Hospitalar de Gaia/Espinho, Rua Conceição Fernandes, 4430-502 Vila Nova de Gaia, Espinho, Portugal; University of Porto Medical School, Porto, Portugal; Division of Imaging Sciences and Biomedical Engineering, The Rayne Institute, Kings College London, St. Thomas’ Hospital, London, UK; Department of Pediatric Cardiology and Intensive Care Medicine, Georg-August University, Göttingen, Germany; Institute for Diagnostic and Interventional Radiology, Georg-August University, Göttingen, Germany; DZHK (German Centre for Cardiovascular Research), Göttingen, Germany; University of Nebraska Medical Center/ Children’s Hospital and Medical Center, Omaha, NE USA; Gastroenterology Department, Centro Hospitalar de Gaia/Espinho, Espinho, Portugal; Department of Cardiology and Pneumology, Georg-August University, Göttingen, Germany

**Keywords:** Liver, Cardiomyopathy, Magnetic resonance, Feature tracking, Strain, Dobutamine stress

## Abstract

**Background:**

Liver cirrhosis has been shown to affect cardiac performance. However cardiac dysfunction may only be revealed under stress conditions. The value of non-invasive stress tests in diagnosing cirrhotic cardiomyopathy is unclear. We sought to investigate the response to pharmacological stimulation with dobutamine in patients with cirrhosis using cardiovascular magnetic resonance.

**Methods:**

Thirty-six patients and eight controls were scanned using a 1.5 T scanner (Siemens Symphony TIM; Siemens, Erlangen, Germany). Conventional volumetric and feature tracking analysis using dedicated software (CMR42; Circle Cardiovascular Imaging Inc, Calgary, Canada and Diogenes MRI; Tomtec; Germany, respectively) were performed at rest and during low to intermediate dose dobutamine stress.

**Results:**

Whilst volumetry based parameters were similar between patients and controls at rest, patients had a smaller increase in cardiac output during stress (*p* = 0.015). Ejection fraction increase was impaired in patients during 10 μg/kg/min dobutamine as compared to controls (6.9 % vs. 16.5 %, *p* = 0.007), but not with 20 μg/kg/min (12.1 % vs. 17.6 %, *p* = 0.12). This was paralleled by an impaired improvement in circumferential strain with low dose (median increase of 14.4 % vs. 30.9 %, *p* = 0.03), but not with intermediate dose dobutamine (median increase of 29.4 % vs. 33.9 %, *p* = 0.54). There was an impaired longitudinal strain increase in patients as compared to controls during low (median increase of 6.6 % vs 28.6 %, *p* < 0.001) and intermediate dose dobutamine (median increase of 2.6%vs, 12.6 % *p* = 0.016). Radial strain response to dobutamine was similar in patients and controls (*p* > 0.05).

**Conclusion:**

Cirrhotic cardiomyopathy is characterized by an impaired cardiac pharmacological response that can be detected with magnetic resonance myocardial stress testing. Deformation analysis parameters may be more sensitive in identifying abnormalities in inotropic response to stress than conventional methods.

## Background

Systolic and diastolic dysfunction, as well as electrophysiological abnormalities have been described in patients with cirrhosis [1–3]. Although previous studies have reported the presence of myocardial dysfunction at resting states [4–6], cirrhotic cardiomyopathy is usually clinically silent and may only be unmasked during physiological or inotropic stress [7].

Pharmacological stress tests using echocardiography or SPECT are often used in cirrhotic patients for diagnosing coronary artery disease before liver transplantation. Although blunted responses of stroke volume and ejection fraction (EF) to dobutamine have also been reported [8], other studies have questioned these findings, particularly in patients with mild disease [9]. Therefore, most authors recommend that dobutamine stress tests should be reserved for excluding ischaemic heart disease before liver transplantation [10].

The inability to increase cardiac output under stress conditions has been associated with the development of hepatorenal syndrome and mortality in cirrhosis [11, 12]; therefore, inotropic incompetence detection may be clinically relevant, identifying patients with a higher risk of complications, which should probably be managed more aggressively.

Cardiovascular magnetic resonance (CMR) has evolved into the reference standard methodology for assessment of cardiac morphology and volumes [13, 14]. Myocardial strain – which may reflect systolic function more accurately than conventional, highly load dependent, indices such as ejection fraction - can also be assessed using CMR, both at rest and during inotropic stimulation with dobutamine [15]. The response of strain to stress has not been previously studied in patients with cirrhosis.

To test the hypothesis that a pharmacological stress test could reveal systolic incompetence in patients with cirrhosis, we performed a comprehensive analysis of systolic function during pharmacological stress, using CMR.

## Methods

The study protocol was approved by the hospital's ethics commitee (Comissão de ética CentroHospitalar de Gaia/espinho EPE) and complies with the declaration of Helsinki. Written informed consent was obtained from all participants.

Thirty-six patients with cirrhosis followed in a hepatology outpatient clinic, able to comply with the instructions during the exam, were recruited and referred to CMR. The diagnosis of cirrhosis was based on clinical, laboratory and ultrasonographic criteria and was also confirmed by liver biopsy in 22 % of the cases. Patients with a known history of hypertension, diabetes, cardiac disease or relevant ECG abnormalities were excluded. Patients with large volume ascites and/or unable to tolerate breath-holding, renal insufficiency (creatinine clearance ≤ 60 ml/min/1.73 m^2^) or standard contraindications to CMR or gadolinium were also excluded.

A group of eight subjects, with similar age and sex distribution as the patient group, without known cardiovascular risk factors, referred to CMR for a different indication (mostly atypical chest pain evaluation) and with a completely normal scan, was used as control.

### CMR acquisition

#### Patient preparation

Patients were instructed to refrain from smoking, coffee, tea, aminophylline, for 24 h before the scan. Beta-blockers were suspended 48 h before the study.

### CMR protocol

Images were acquired using a 1.5 T scanner (Siemens Symphony TIM; Siemens, Erlangen, Germany) with a 6-channel anterior chest coil and spinal coils within the gantry table.

### Cine imaging

After scout images, cine images using a retrospective ECG-gated balanced steady state free-precession sequence (TR 3.0 ms, TE 1.3 ms, flip angle < 90°) were acquired during brief periods of end-expiratory breath-hold. Two-, four and three-chamber orientations, as well as multiple equidistant short-axis planes (slice thickness 8 mm; gap 2 mm) allowing coverage of the entire cardiac volume were performed. Thirty phases were obtained per cardiac cycle.

For dobutamine stress imaging, three long-axis and three short-axis slices (basal, mid-ventricular and apical) were acquired, in order to cover 16 myocardial segments [16]. Dobutamine was infused intravenously at 3-min stages at doses of 10 and 20 μg/kg/min. Repeat short-axis images as well as long-axis images were acquired at the end of each stage. During dobutamine infusion, patient symptoms, heart rate, blood pressure, and electrocardiogram were monitored.

### Aortic flow imaging

Aortic flow was measured using phase contrast gradient echo pulse sequence with one-direction “through-plane” motion-encoding (slice thickness 5 mm; FOV 320 × 320 mm^2^, in-plane resolution ≤1 mm, TR/TE = 5.9/3.0 ms, flip angle 22°, bandwidth ~350 Hz/pixel), centered in ascending aorta and aligned orthogonally to the expected main blood flow direction in two spatial directions, at the level of the pulmonary bifurcation. Velocity encoding sensitivity (Venc) was adapted to the expected velocities (typically 150 for the rest images and 300 during the dobutamine-stress acquisitions). Thirty frames were acquired per cardiac cycle using a free-breathing technique with three excitations per k-space line.

### Perfusion imaging

Our protocol for stress perfusion imaging has been previously described [17]. Maximal hyperemia was achieved with intravenous adenosine (140 μg.kg − 1.min − 1) infusion for 5 min. Within the last 2 min of infusion, an intravenous bolus of 0.07 mmol/Kg of gadobutrol (Gadovist, Bayer HealthCare Pharmaceuticals, Berlin, Germany), was injected. Three short-axis slices (basal, mid-ventricular and apical) were imaged during the first pass of the bolus of gadolinium using a gradient echo pulse sequence with a single saturation pre-pulse per R–R interval shared over the three slices. Typical sequence parameters were: echo time, 1.18 ms; repetition time, 192 ms; inversion time, 110 ms; flip angle, 12°; slice thickness, 10 mm; field of view, 290–460 mm; matrix, 192 × 128 mm; in-plane spatial resolution, 1.5–2.4 mm [2]; bandwidth, 789 Hz per pixel. Patients were asked to hold their breath on full expiration for the duration of the first pass of the gadolinium bolus.

### Late gadolinium enhancement

Late gadolinium-enhancement (LGE) was assessed using a gradient-recalled phase-sensitive inversion-recovery (PSIR) sequence (TR 46 ms, TE 3.4 msec, flip angle 15°, IR time 280–360 msec) ≥10 min after the administration of 0.2 mmol/kg of gadobutrol.

### CMR analysis

Images were anonymized and analysis was performed by operators blinded to clinical data.

A commercially available software (CMR42; Circle Cardiovascular Imaging Inc., Calgary, Canada) was used to assess left and right ventricular volumes and function, from the short-axis cine images stack. Left ventricular ejection fraction (EF) during stress was derived from two long axis and one short axis; for comparison the same method was also used to calculate resting EF. Phase-contrast pulse sequences at rest and peak dobutamine dose were analyzed with the same software, to determine cardiac output.

Feature tracking (FT), a technique analogous to echocardiographic speckle tracking, which allows tracking of tissue voxel motion of CMR cine images [18–21] was used to assess left ventricular strain. Four-, two- and three-chamber views were used to calculate longitudinal strain. Radial strain and circumferential strain were derived from the three short-axis planes. For each parameter three repeated measurements were performed and subsequently averaged. Global longitudinal strain (GLS), global radial strain (GRS) and global circumferential strain (GCS) were defined as the mean strain of the three individual planes. Measurements were performed at rest and at each stage of dobutamine infusion.

For the stress perfusion analysis, perfusion defects were defined as subendocardial or transmural visually dark myocardial areas when compared with remote healthy myocardium, persisting for at least 10 frames.

### Reproducibility

Reproducibility of FT derived strain was assessed in 10 randomly selected subjects. For intraobserver variability, the same operator repeated the measurements, more than 4 weeks after the initial analysis. For interobserver variability, a second operator re-analysed the images.

### Statistical analysis

Data were stored and analyzed using IBM SPSS Statistics, Version 20.0 (IBM Corp., Armonk, NY, USA). Results are presented as median (25th–75th percentile) for quantitative variables and as n (%) for categorical variables. A significance level of 5 % was used.

The Mann–Whitney test was used to evaluate differences in continuous variables between groups. The Chi-squared test was used to compare proportions. Spearman’s coefficient was used to test correlations. Bland–Altman analysis was performed for reproducibility testing.

## Results

Clinical characteristics and laboratorial characteristics of patients and controls are shown in Table 1. Most patients (*n* = 27, 75 %) were in Child-Pugh class A, eight patients (17.8 %) were in class B and only one patient was in class C.

**Table 1:** Clinical and laboratorial characteristics of patients and controls

	Patients (*n* = 36)	Controls (*n* = 8)	p
Age	54 (48-61)	52 (45-54)	0.12
Male gender (n, %)	30 (83.3)	5 (62.5)	0.33
Cirrhosis aetiology			
Alcoholic (n, %)	21 (58.3)		
Viric (n, %)	10 (27.8)		
Other (n, %)	5 (13.9)		
Child-Pugh score	5 (5-7)		
MELD score	9 (7-11)		
Diuretic use (n, %)	7 (19.4 %)		
Heart rate	72 (58-78)	69 (51-72)	0.20
Mean blood pressure	98 (88-106)	100 (99-104)	0.64
Blood analysis			
Haemoglobin (g/dL)	13.4 (11.5-15.3)	14.4 (13.6-15.5)	0.21
Platelet count (×10^9^/L)	101 (76-142)	225 (182-256)	<0.001
Creatinine (mg/dL)	0.63 (0.52-0.79)	0.74 (0.49-0.95)	0.66
Sodium (mEq/L)	139 (137-141)	142 (140-143)	0.035
Total bilirubin (mg/dL)	0.92 (0.61-1.30)	0.34 (0.22-0.48)	<0.001
Albumin (g/dL)	4.1 (3.6-4.5)	4.6 (4.5-4.9)	0.005
NT-ProBNP (pg/mL)	58 (30-140)	32 (22-53)	0.20
CRP (mg/dL)	0.25 (0.11-0.52)	0.16 (0.06-0.38)	0.42
INR	1.2 (1.1-1.3)	1.0 (0.9-1.1)	0.001

Results are presented as median (25th–75th percentile) for quantitative variables

*CRP* C-Reactive Protein, *INR* International Normalized Ratio, *MELD* Model for End-Stage Liver Disease, *NT-proBNP* N-terminal pro–B-type natriuretic peptide

CMR –derived morphological and functional parameters were similar at rest in patients and controls (Table 2). We found no differences in resting GLS, GCS or GRS between patients and controls. Child-Pugh class A patients had a trend towards lower left atrial volume compared to patients with more severe (class B and C) disease [43.3 ml/m^2^ (35.4-49.5) vs 47.6 ml/m^2^ (44.1-56.7); *p* = 0.08]. No differences in any of the other parameters were found, in resting conditions, between these two groups of patients.

**Table 2:** CMR parameters at rest of patients and controls

	Patients (*n* = 36)	Controls (*n* = 8)	p
Left atrial volume (ml/m^2^)	44.9 (36.1-51.9)	44.2 (37.5-49.4)	0.92
Right atrial area (cm^2^)	21 (18-23)	22 (20-25)	0.35
Left ventricular diastolic volume (ml/m^2^)	75.1 (65.1-92.1)	87.7 (74.1-94.6)	0.27
Left ventricular systolic volume (ml/m^2^)	24.4 (19.1-28.9)	28.0 (23.2-33.3)	0.19
Left ventricular ejection fraction (%)	67 (64-72)	66.0 (64-70)	0.66
Left ventricular mass (g/m^2^)	54.7 (46.7-62.0)	55.7 (45.7-63.5)	0.96
Right ventricular diastolic volume (ml/m^2^)	84.5 (67.9-92.2)	84.1 (70.5-97.7)	0.46
Right ventricular ejection fraction (%)	57 (52–62)	58 (56-61)	0.46
Cardiac output (l/min)	6.5 (5.1-7.9)	6.1 (5.1-6.6)	0.74
GLS (%)	−18.9 (−16.0 to −20.5)	−19.0 (−16.1 to −20.6)	0.96
Time to Peak GLS (ms)	263 (206-317)	253 (225-281)	0.96
GCS (%)	−27.5 (−24.1 to −30.6)	−27.7 (−24.9 to −30.1)	0.84
Time to Peak GCS (ms)	264 (208-315)	223 (216–316)	0.71
GRS (%)	33.9 (25.4-39.1)	39.1 (34.8-41.8)	0.80
Time to Peak GRS (ms)	275 (216–308)	223 (215-303)	0.36

Results are presented as median (25th–75th percentile)

*GCS* Global Circumferential Strain, *GLS* Global Longitudinal Strain, *GRS* Global Radial Strain

### Dobutamine stress

**Fig. 1:**
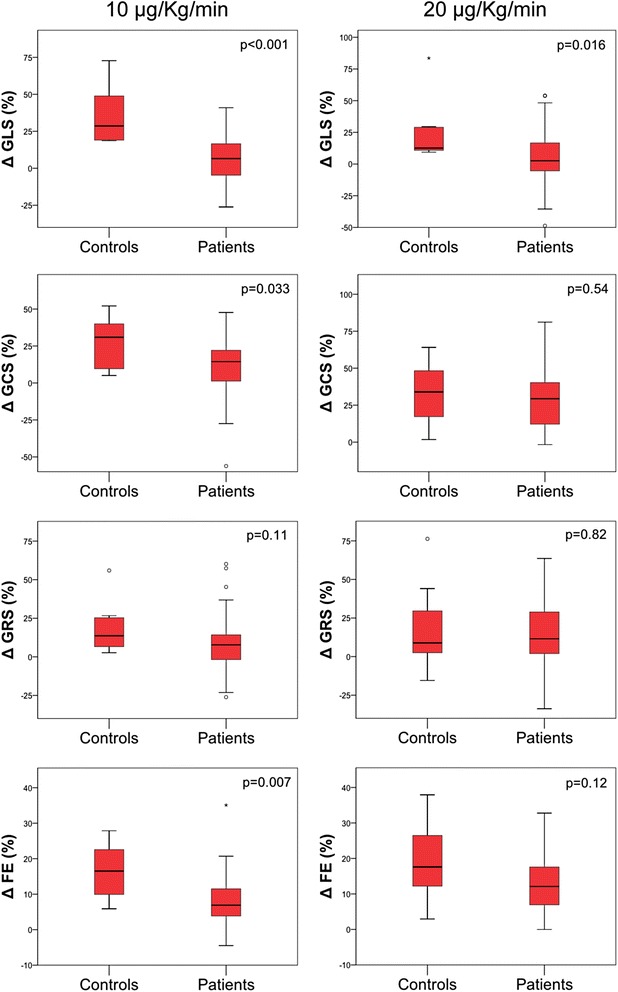
Strain and ejection fraction response to pharmacological stress. Percentual variation of strain parameters and ejection fraction with 10 μg/Kg/min and 20 μg/Kg/min of dobutamine in patients and controls. GLS – global longitudinal strain; GCS – global circumferential strain; GRS – global radial strain; EF – ejection fraction

**Fig. 2:**
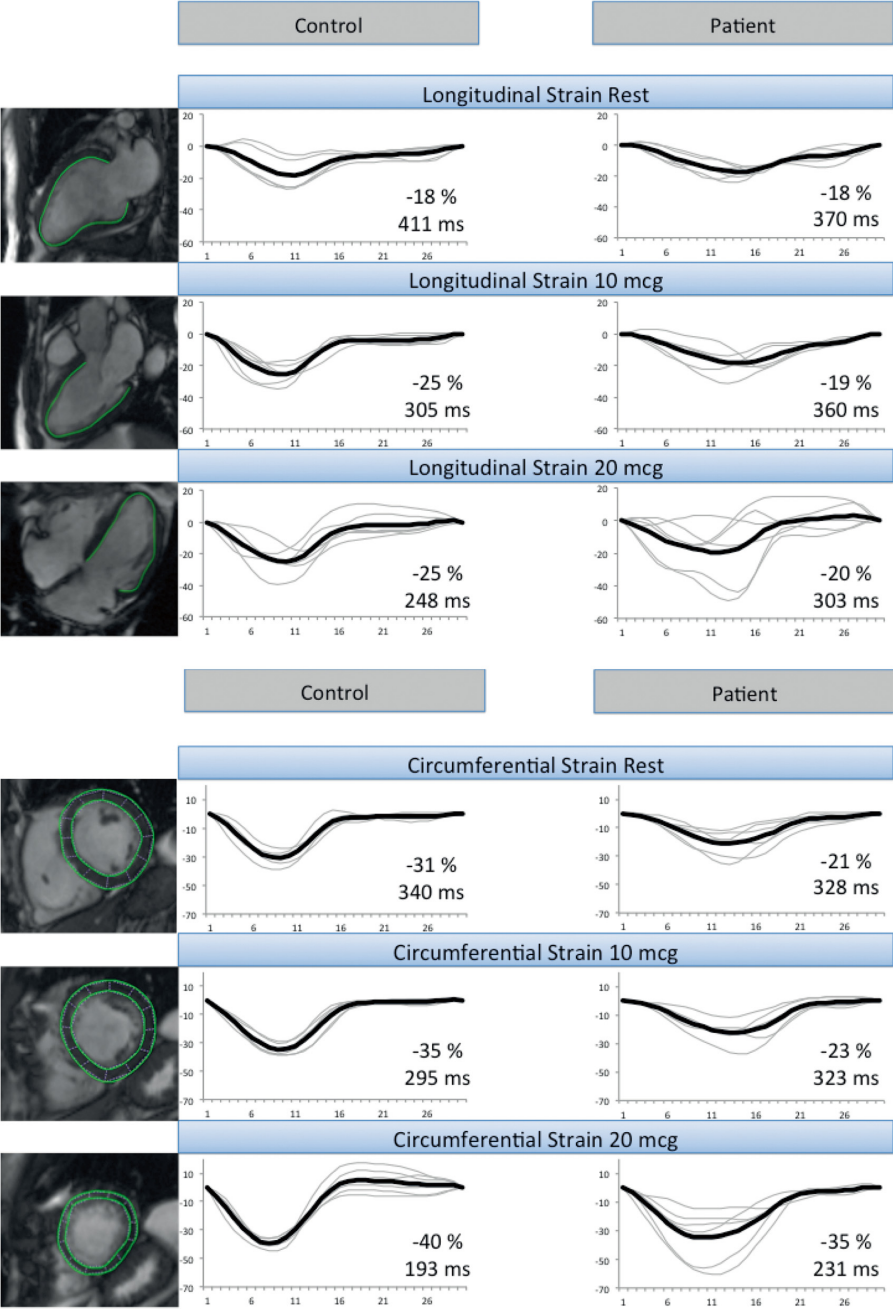
Blunted response of strain to dobutamine. Representative example of the blunted response of longitudinal and circumferential strain to dobutamine in a patient, as compared to a control. Values written in the diagrams correspond to peak strain (%) and time to peak strain (ms), respectively

The response of hemodynamic and strain parameters to increasing doses of dobutamine is shown in Table 3 and Figs. 1 and 2. Compared to controls, patients had a smaller increase of stroke volume and cardiac output during dobutamine perfusion. There was no difference in heart rate response to stress in the two groups. The increase in EF was lower in patients than in controls at the dose of 10 μg/kg/min of dobutamine (median percentual increase of 6.9 % (3.5-12.1) vs 16.5 % (8.5-23.3), *p* = 0.007), but not at 20 μg/kg/min (median percentual increase of 12.1 % (6.8-17.9) vs 17.6 % (10.4-28.0), *p* = 0.12). The improvement in GLS during the infusion was significantly lower in patients compared to controls, both at 10 μg/kg/min (median percentual increase of 28.6 % (6.6 % (−5.8-17.0) vs 18.9-54.4), p < 0.001) and at 20 μg/kg/min (median percentual increase of 2.6 % (−5.5-16.7) vs 12.6 % (10.4-29.2), *p* = 0.016). Global circumferential strain increased less significantly in patients as compared to controls at 10 μg/kg/min (median percentual increase of 14.4 % (0.6-22.9) vs 30.9 % (8.6-41.5), *p* = 0.03); the response of GCS to 20 μg/kg/min of dobutamine was not significantly different between the two groups (median percentual increase of 29.4 % (10.9-41.1) vs 33.9 % (16.7-48.5), p = 0.54). The response of GRS to pharmacological stress was not different in patients versus controls (median percentual increase of 7.7 % (−2.4-15.2) vs 13.6 % (5.7-26), *p* = 0.11 at 10 μg/kg/min of dobutamine and 4.3 % (0.6-8.1) vs 3.1 % (−0.2-9.3), *p* = 0.82 at 20 μg/kg/min of dobutamine).

**Table 3:** Hemodynamic and strain response to dobutamine

	Patients			Controls			p
	Rest	10 μg/Kg/min	20 μg/Kg/min	Rest	10 μg/Kg/min	20 μg/Kg/min	
Heart Rate (beats/min)	72 (58-78)	75 (63-84)	94 (75-111)	69 (51-72)	65 (53-91)	90 (72-118)	
Δ Heart Rate (beats/min)		3 (−2-10)			3 (−5-14)		0.94
			24 (5-34)			25 (18-42)	0.44
Stroke volume (ml)	92 (80-110)		95 (73-113)	93 (71-115)		117 (86-144)	
Δ Stroke volume (ml)			−1.0 (−15-18)			18 (4-29)	0.037
Cardiac Output (l/min)	6.5 (5.1-7.9)		9.2 (7.0-10.4)	6.1 (5.1-6.6)		9.4 (8.6-11.5)	
Δ Cardiac Output (l/min)			2.2 (1.7-3.2)			3.8 (3.4-4.9)	0.015
Ejection Fraction (%)^a^	66 (60-69)	71 (65-74)	73 (70-76)	63 (61-67)	73 (72-76)	75 (73-79)	
Δ Ejection Fraction (%)		4 (2-8)			10 (6-14)		0.006
			8 (5-11)			11 (7-17)	0.08
GLS (%)	−18.9 (−16.0 to −20.5)	−19.7 (−15.9 to −22.4)	−19.5 (−16.6 to –21.6)	−19.0 (−16.1 to −20.6)	−23.4 (−22.1 to –26.7)	−21.8 (−19.9 to –23.6)	
Δ GLS (%)		−1.4 (1.0 to −3.1)			−5.9 (−3.6 to −8.2)		<0.001
			−0.6 (1.0 to –3.0)			−2.5 (−2.3 to −5.1)	0.018
GCS (%)	−27.5 (−24.1 to −30.6)	−31.2 (−25.6 to −35.7)	−35.7 (−30.3 to −39.3)	--27.7 (−24.9 to −30.1)	−34.7 (−32.2 to −36.3)	−36.1 (−34.8 to −37.3)	
Δ GCS (%)		−4.0 (−0.1 to −5.8)			−8.3 (−2.6 to −10.5)		0.04
			−7.6 (−3.7 to −11.0)			−9.1 (−5.0 to −13.2)	0.48
GRS (%)	33.9 (25.4-39.1)	35.6 (28.9-41.8)	38.1 (29.7-43.1)	31.8 (28.5-40.0)	39.1 (34.8-41.8)	36.3 (33.9-41.4)	
Δ GRS (%)		1.9 (−0.9-5.3)			4.2 (2.2-8.2)		0.10
			4.2 (0.6-8.1)			3.1 (−0.2-9.3)	0.79

Results are presented as median (25th–75th percentile)

*GLS* global longitudinal strain, *GCG* global circumferential strain, *GRS* global radial strain, Δ absolute variation from baseline

^a^Derived from two long axis and one short axis

We found no differences in the response of hemodynamic and strain parameters to dobutamine between patients in Child-Pugh class A versus the others or between patients on diuretics versus patients without prior diuretic use.

There was a correlation between the percentual change of GLS and the percentual change in left ventricular stroke volume (Spearman’s rho = 0.42, *p* = 0.007). Changes in stroke volume and GCS (Spearman’s rho = 0.11, *p* = 0.48) or GRS (Spearman’s rho = 0.07, *p* = 0.69) were not correlated.

### Stress perfusion and late gadolinium enhancement

No perfusion abnormalities were detected in any subject during adenosine stress. There was no LGE in patients and controls.

### Reproducibility

The mean differences and confidence intervals between repeated strain measurements are displayed in Table 4. Reproducibility was similar at rest and with dobutamine stress. Variability was higher for GRS than for GLS and GCS.

**Table 4:** Variability of different strain parameters at rest and during dobutamine stress

	Intraobserver variability			Interobserver variability		
Parameter	Mean difference (%)	95 % CI	p	Mean difference (%)	95 % CI	p
Rest						
GLS	0.17	−0.83-0.87	0.96	0.78	−0.57-2.13	0.23
GRS	0.92	−3.11-3.29	0.96	3,64	0.96-6.32	0.01
GCS	0.47	−0.21-1.15	0.24	0.39	−0.35-1.13	0.26
Dobutamine 10 μg/kg/min						
GLS	0.49	−1.01-2.0	0.29	1.12	0-2.24	0.05
GRS	0.64	−3.01-4.29	0.68	3.77	0.20-7.35	0.04
GCS	0.45	−0.51-1.41	0.31	0.39	−0.49-1.27	0.34
Dobutamine 20 μg/kg/min						
GLS	0.56	−0.53-1.66	0.17	0.77	−0.36-1.91	0.16
GRS	2.02	0.09-3.94	0.04	2.96	−0.70-6.63	0.10
GCS	0.39	−0.39-1.18	0.17	0.94	−0.34-2.22	0.13

*GLS* Global Longitudinal Strain, *GRS* Global Radial Strain, *GCS* Global Circumferential Strain

## Discussion

Our results suggest that a dobutamine stress test can be useful in revealing systolic abnormalities in patients with mild cirrhosis. Using cardiovascular magnetic resonance derived strain, we have shown that, during low- to intermediate dose dobutamine stimulation, patients with cirrhosis had a smaller improvement in longitudinal and a delayed increase in circumferential strain compared to controls. This is, to the best of our knowledge, the first study to evaluate myocardial deformation during stress in cirrhosis.

Subendocardial fibre damage with consequent left ventricular longitudinal function impairment are usually the first manifestations of myocardial disease [22–24]. Longitudinal dysfunction was previously documented in cirrhosis, at rest [5, 6], using echocardiography. In the present study, we could not find differences in any CMR parameters at rest between patients and controls. This may be related to the early stage of disease of the majority of the patients. Patients exhibited a blunted response of GLS to dobutamine, compared to controls. The normal response of strain to dobutamine is an initial increment followed by a plateau or decrement (when filling is reduced by increased heart-rate) [25, 26]. Our results are in line with this pattern in controls but not in the patient group. This suggests that, while in controls, the maximal inotropic effect of dobutamine is achieved with the lower dose (the higher dose having mainly a positive chronotropic effect), patients may also have a delayed response of longitudinal strain to dobutamine, with some strain still further developing at 20 μg/kg/min. Several abnormalities in cardiomyocyte structure and function - including decreased density and down-regulation of beta-adrenergic receptors and impaired intracellular signaling pathways - have been described in animal models of cirrhosis [27, 28] and may account for our findings.

Compared to controls, patients also had a smaller increase of GCS with 10 μg/kg/min of dobutamine; the response of GCS to 20 μg/kg/min did not differ between the two groups. This also suggests a delayed response of circumferential strain to inotropic stimuli in cirrhosis with patients requiring higher doses of dobutamine (or more time) to equalize with the controls. Ejection fraction showed a similar behaviour: patients had a smaller improvement at the dose of 10 μg/kg/min of dobutamine but no differences were found between the groups at 20 μg/kg/min. The response of GRS to dobutamine was not different in patients and controls; this is in line with previous studies in ischemic heart disease patients, which have reported radial strain to be the last component of myocardial mechanics to be affected by ischemia [29].

Although previous studies have reported an abnormal cardiac response to exercise or pharmacological stress in cirrhotic patients using echocardiography and SPECT [7, 30, 31], the role of dobutamine stress testing in diagnosing cirrhotic cardiomyopathy is still a matter of debate, since its ability to detect abnormalities – mainly changes in volumes and EF – has been inconsistent using these imaging modalities [8, 9, 32]. The role of dobutamine stress MRI in cirrhosis has not been previously studied. Myocardial deformation analysis in this setting has also never been reported, either with CMR or echocardiography (probably because its feasibility under stress with the latter may be limited). Unlike echo, CMR does not depend on a good “acoustic window” for image acquisition, and its feasibility in quantifying strain during dobutamine stress has been demonstrated [15, 33]. Our findings may explain previous negative results [9] since preserved radial mechanics and a normal response of circumferential strain to higher doses of dobutamine, may contribute to a normal EF response during these doses, despite the smaller longitudinal strain increase.

Cardiac output increased less significantly in patients than in controls. This seems to be explained by the higher increase in stroke volume found in controls, since heart rate variation was similar in the two groups. We also found a significant correlation between the dobutamine-induced changes of GLS and stroke volume. According to these observations, inotropic incompetence, which has been reported as a feature of cirrhotic cardiomyopathy may be at least partially explained by longitudinal myocardial dysfunction. Our findings may be clinically relevant since the inability to increase cardiac output under stress conditions (such as infection or haemorrhage) may play a role in the development of hepatorenal syndrome, influencing mortality in patients with cirrhosis [11, 12]. Hence, patients exhibiting an abnormal inotropic response under pharmacological stress may need a closer follow-up and a more aggressive management of complications. However, since other studies have failed to establish a relation between cardiac dysfunction and prognosis [34–36] and we did not assess prognosis, this hypothesis remains speculative, and warrants future research.

Resting hemodynamic conditions may influence inotropic response to pharmacological stress, and our results might have been related to differences in volemia (particularly in the presence of diuretics), or neuro-humoral stimulation. However, only seven patients were on diuretics and we could not find any differences in inotropic response between them and the other patients; we also failed to find differences in cardiac chambers size, resting heart rate, blood pressure or cardiac output between patients and controls. Taken altogether, these findings argue against an effect of different basal hemodynamics on our results.

Under adenosine stress, we did not detect ischemia, which could have influenced inotropic response to dobutamine, in any subject. Although a quantitative perfusion analysis was not performed, our methodology has been shown to be highly accurate in detecting functionally significant coronary artery disease [17].

In contrast with a previous study [37], we did not find LGE in any patient. The difference in disease severity between the two studies probably accounts for these findings since myocardial fibrosis may only be detectable in more advanced disease states as a result of the chronic activation of the renin-angiotensin-aldosterone system.

### Limitations

This is a single center study performed in patients mainly with alcoholic cirrhosis and mild disease. Since we have excluded patients that would not be able to comply with the instructions or tolerate the breath holding at rest our results may not be generalized to all patients with cirrhosis. Furthermore, we cannot exclude the occurrence of type two errors, due to the small size of the control group.

We aimed to evaluate the contractile response to dobutamine stress with the maximum extent of inotropic response expected with doses of 10–20 μg/kg/min of dobutamine [38]. However we cannot exclude that a full test (40 μg/kg/min) would have potentially added valuable information despite the fact that we didn’t observe significant perfusion defects with adenosine stress.

Due to time constraints, no full cine short axis stacks for volumetric analysis were obtained during stress. Additionally, it would have been interesting to evaluate diastolic function, particularly under stress. Although diastolic strain rate can be computed with feature tracking, the relatively low temporal resolution of cine imaging (further reduced at faster heart rates during stress), may limit its feasibility.

There is no widely accepted gold-standard method to diagnose cirrhotic cardiomyopathy; on the other hand, there are no well-established normal values of CMR-derived strain parameters at rest and under pharmacological stress. Hence, the diagnostic accuracy of our methodology cannot be objectively determined and definitive cut-offs cannot be provided.

We could not perform a T1-mapping analysis, which might have allowed us to detect the presence of diffuse myocardial fibrosis.

## Conclusions

Patients with cirrhosis show inotropic incompetence to pharmacological stress, due to intrinsic myocardial dysfunction.

CMR with myocardial deformation analysis may be a sensitive diagnostic tool to identify abnormal inotropic responses to stress already present at early disease states, which may be difficult to detect with other non-invasive imaging modalities. The significance of this impaired response to pharmacological stress in cirrhotic cardiomyopathy and its prognostic implications should be further explored in future prospective clinical investigations.

## Abbreviations

SPECT: Single-photon emission computed tomography
EF: Ejection fraction
CMR: Cardiovascular magnetic resonance
FT: Feature tracking
GLS: Global longitudinal strain
GRS: Global radial strain
GCS: Global circumferential strain
LGE: Late gadolinium enhancement
